# Interpreting malaria age-prevalence and incidence curves: a simulation study of the effects of different types of heterogeneity

**DOI:** 10.1186/1475-2875-9-132

**Published:** 2010-05-17

**Authors:** Amanda Ross, Thomas Smith

**Affiliations:** 1 Department of Epidemiology and Public Health, Swiss Tropical and Public Health Institute, Basel, Switzerland; 2 University of Basel, Basel, Switzerland

## Abstract

**Background:**

Individuals in a malaria endemic community differ from one another. Many of these differences, such as heterogeneities in transmission or treatment-seeking behaviour, affect malaria epidemiology. The different kinds of heterogeneity are likely to be correlated. Little is known about their impact on the shape of age-prevalence and incidence curves. In this study, the effects of heterogeneity in transmission, treatment-seeking and risk of co-morbidity were simulated.

**Methods:**

Simple patterns of heterogeneity were incorporated into a comprehensive individual-based model of *Plasmodium falciparum *malaria epidemiology. The different types of heterogeneity were systematically simulated individually, and in independent and co-varying pairs. The effects on age-curves for parasite prevalence, uncomplicated and severe episodes, direct and indirect mortality and first-line treatments and hospital admissions were examined.

**Results:**

Different heterogeneities affected different outcomes with large effects reserved for outcomes which are directly affected by the action of the heterogeneity rather than via feedback on acquired immunity or fever thresholds. Transmission heterogeneity affected the age-curves for all outcomes. The peak parasite prevalence was reduced and all age-incidence curves crossed those of the reference scenario with a lower incidence in younger children and higher in older age-groups. Heterogeneity in the probability of seeking treatment reduced the peak incidence of first-line treatment and hospital admissions. Heterogeneity in co-morbidity risk showed little overall effect, but high and low values cancelled out for outcomes directly affected by its action. Independently varying pairs of heterogeneities produced additive effects. More variable results were produced for co-varying heterogeneities, with striking differences compared to independent pairs for some outcomes which were affected by both heterogeneities individually.

**Conclusions:**

Different kinds of heterogeneity both have different effects and affect different outcomes. Patterns of co-variation are also important. Alongside the absolute levels of different factors affecting age-curves, patterns of heterogeneity should be considered when parameterizing or validating models, interpreting data and inferring from one outcome to another.

## Background

A myriad of differences between individuals in a community affect the epidemiology of malaria. These differences arise in roughly four ways: heterogeneity of transmission; biological heterogeneity of the host in susceptibility and response to malaria infection[1-4]; heterogeneity in host behaviour, including quality of housing, use and knowledge of protective measures such as insecticide-treated nets (ITN) and treatment-seeking strategies; and heterogeneity in the risk of co-morbidity and malnutrition.

The different sources of heterogeneity are unlikely to be independent. A factor, such as socio-economic status (SES), may be associated with several different heterogeneities. SES can influence transmission intensity through quality of housing, knowledge of protection measures and use of ITNs [5-8]. Risks of co-morbidity and malnutrition may be associated with SES [9,10]. SES may influence treatment-seeking behaviour [7]. Amongst those caring for children, treatment-seeking [7,11-13] and knowledge of danger signs [10,11] were worse in poorer families. Such inequalities tend to persist over time [14]. Thus, covariance between different types of heterogeneity is plausible although the degree to which it occurs is likely to vary between sites.

Differences among individuals can lead to important patterns at the community level [15,16]. Different types of heterogeneity may have implications for both the interpretation of field data and for formulating and calibrating models of the effects of interventions, or alternatively may have little impact and could be safely ignored. Co-variation may describe sub-groups within a population, who perhaps respond differently to an intervention (for example [17]) or who are reached neither by the health system nor by any of several interventions.

Assessing the effects of different kinds of heterogeneity using field datasets is not practical due to difficulties in collecting the relevant data and isolating the effects of each of the heterogeneities. Therefore, heterogeneity has been investigated using mathematical models. There are few examples of heterogeneity modelling studies specific to malaria (for example [15,18-25]) and very few incorporating multiple types of heterogeneity [23,24]. One reason may have been the models themselves. To study the effects of multiple types of heterogeneity, a model must be sufficiently comprehensive, dynamic to allow secondary effects, and be able to easily incorporate different kinds of heterogeneity.

A recently published model of *Plasmodium falciparum *malaria epidemiology [26] satisfies these criteria. It includes processes for infection, parasitaemia, acquired immunity, infectivity, morbidity and mortality and case-management. It is dynamic, allowing feedback effects such as the effects of high treatment coverage on transmission intensity. It is also individual-based, which provides a flexible framework to conveniently incorporate heterogeneity [27-29].

This model is adopted as a base to investigate the effects of three different types of heterogeneity: transmission (including host behaviours such as bed net use and housing), co-morbidity (as a trigger for severe malaria and indirect mortality and reflecting the individual's general health and nutrition) and treatment-seeking (the chances of obtaining effective treatment). These heterogeneities are simulated singly, and in independent and co-varying pairs. This paper focuses on the effects of heterogeneity on fundamental measures of the malaria burden, age-prevalence and incidence curves. These curves are known to vary by the severity of the outcome, degree of seasonality and the overall levels of transmission intensity (reviewed in [30]).

## Methods

### Base model

The stochastic model of malaria epidemiology has been described in detail elsewhere [26]. Briefly, a simulated population of humans are updated at each five-day time-step via components representing new infections, parasite densities, acquired immunity, uncomplicated and severe episodes, direct and indirect malaria mortality, and infectivity to mosquitoes. The course of parasite densities over an infection are described by averaged empirical data [31]. Immunity to asexual parasites is derived from a combination of cumulative exposure to both inoculations and parasite densities, and maternal immunity [31]. The inclusion of acquired immunity allows us to model potential effects of heterogeneity on immunity through exposure being averted or increased. The probability of a clinical attack of malaria depends on the current parasite density and a pyrogenic threshold [32]. The pyrogenic threshold responds dynamically to recent parasite load, increasing or saturating through exposure to parasites and decaying with time, and thus is individual- and time-specific. Severe malaria can arise in two ways, either as a result of overwhelming parasite density or through uncomplicated malaria with concurrent non-malaria co-morbidity [33]. Treatment of malaria is based on the case management model of Tediosi and colleagues [34] which involves implementing case management trees for severe cases, and uncomplicated cases with and without recent history of treatment. In the current simulations, we assumed first-line treatment of uncomplicated episodes to be an effective short-acting drug such as artemisinin-combination therapy (ACT) with an average probability of treatment at any one five-day time step of 20%. This value is higher than previously used [34-37]; it was raised arbitrarily to allow the effects of treatment heterogeneity to show. The treatment probability for severe cases warranting hospital admission was 48% [33]. Mortality in this model can be either direct (following severe malaria) or indirect (uncomplicated malaria in conjunction with co-morbidity, or during the neonatal period as a result of maternal infection) [33]. This co-morbidity is simulated as occurring with an age-dependent probability described by a hyperbolic function [33].

The parameter values for the base model were estimated by fitting to data from a total of 61 malaria field studies of various different aspects of malaria epidemiology [38], and are reported elsewhere [37].

### Types of heterogeneity

Some individual differences are inherent to the base model and arise due to host age and an individual's propensity for high parasite densities [31]. In addition, stochasticity occurs throughout the model.

**Figure 1:**
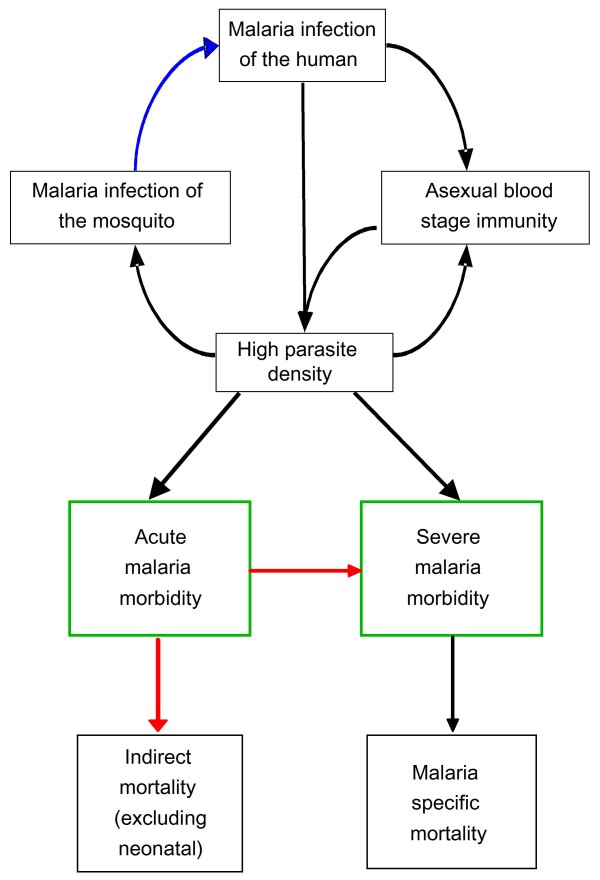
**Model processes and the types of heterogeneity**. Blue arrow: Transmission heterogeneity affects an individual's availability to mosquitoes which is represented in the base model as an age-dependent function. Red arrows: Heterogeneity in the risk of co-morbidity affects the probability of a non-malaria illness or poor nutritional status which, in conjunction with an acute malaria episode, leads to either severe malaria or to an indirect death. In the base model, the risks of these two sets of co-morbidity are represented by two age-dependent functions. Green boxes: Treatment-seeking heterogeneity affects the probabilities that effective treatment is sought for either a fever or for a severe episode per five day time step. In the base model, these probabilities are represented by two constants.

We extended the base model by introducing an additional three types of heterogeneity: (i) transmission, (ii) co-morbidity and (iii) treatment-seeking probability. This study does not consider heterogeneity in the biological response of the host (except for the host dependent heterogeneity in parasite densities incorporated into the base model). The model processes are shown in Figure 1. Each simulated individual is assigned a status for each of the three kinds of heterogeneity at birth, which they carry throughout their life. The structure of the heterogeneities is that 50% of the population are assigned to each of the high and low status categories, with baseline values multiplied by either 1.8 or 0.2. Hence, when assuming heterogeneity in transmission, the age-adjusted entomological inoculation rate (EIR) for individual *i *at time *t *takes the value: *E*_*L*_(*i*,*t*) = 0.2*E*_*a*_(*i*,*t*) for the low exposure half of the population, where *E*_*a*_(*i*, *t*) is the age-adjusted EIR in the base model, and *E*_*H*_(*i*,*t*) = 1.8*E*_*a*_(*i*,*t*) in the high exposure half of the population. Similarly, in models of treatment heterogeneity, the probability that either an uncomplicated or severe attack is treated during any one simulation step takes a value of 1.8 (or 0.2) times that in the base model; in models of heterogeneous co-morbidity, two age-dependent probabilities are multiplied by 1.8 (or 0.2), the probability that a clinical attack becomes severe due to co-morbidity in any one five-day time step and the probability that, following a malaria episode which weakens the host, a non-malaria illness occurs leading to an indirect death.

In simulations of co-varying heterogeneities, the same 50% of the population are at greater risk on each of the three dimensions. Thus low access to treatment is paired with high numbers of infective bites and high risk of co-morbidity.

The present analyses make use of estimates of the remaining parameters in the model derived assuming no heterogeneity in the three variables that are the subject of this study [38]. Results of re-estimation of these parameters conditional on different assumptions about heterogeneity will be reported elsewhere.

### Scenarios simulated

A reference scenario was defined using the input values in Table 1. This study does not report simulations of interventions other than case-management. The three types of heterogeneity were added to the base model and their effects systematically simulated singly, in independent pairs, and in co-varying pairs. The outcomes were parasite prevalence, uncomplicated and severe clinical episodes, direct and indirect mortality, first-line treatments and hospital admissions. The age-prevalence and incidence curves were described using the age at, and height of, the peak. To investigate the effect of transmission intensity on the effects of heterogeneity, this set of simulations was repeated for settings with 1, 6 and 200 infectious bites per person per year. The seasonality in all scenarios followed that of Namawala, Tanzania [39] (Table 1). Average annual transmission levels were held constant so that effects were not simply due to changes in transmission.

**Table 1:** Reference scenario characteristics.

Input	Value
Transmission intensity	21 infected bites per year per adult ^†^
Seasonality	Perennial with seasonal peaks (Namawala, Tanzania [39])^†^
Treatment-seeking	0.2 per fever-5 day interval ^‡^
Treatment drug	ACT (clears all infections)^†^
Demographic age-distribution	Ifakara, Tanzania [53]^†^

^† ^These values were used in previous simulations [35-37]

^‡ ^This value is higher than previous simulations to allow the effect of treatment heterogeneity to show clearly

## Results

### The effect of single heterogeneities

#### Transmission

**Figure 2:**
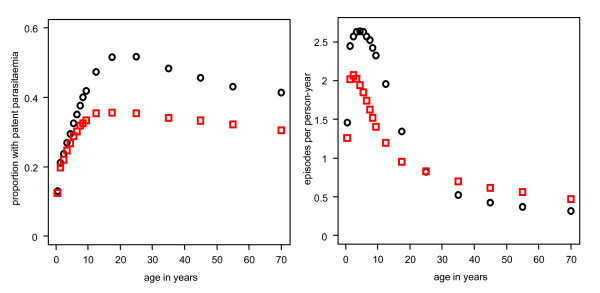
**Predicted effect of heterogeneity in transmission on (a) prevalence and (b) incidence of uncomplicated episodes**. Black circles = reference scenario defined in Table 1. Red squares = with heterogeneous transmission.

Heterogeneity in transmission affected all of the outcomes (Additional File 1: Table S1). The predicted prevalence of parasitaemia was reduced (Figure 2a). For the other outcomes, the incidence rates were reduced in younger age groups compared to the reference scenario and increased for older ages (Figure 2b).

#### Co-morbidity risk

**Figure 3:**
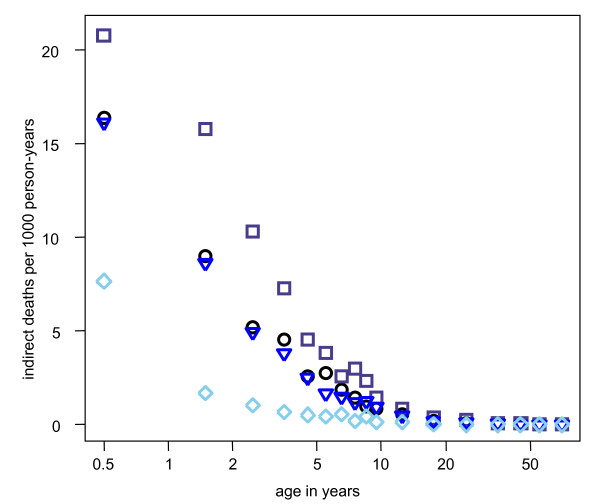
**Predicted effect of co-morbidity risk on indirect malaria mortality**. Black circles = reference scenario as defined in Table 1; blue triangles = heterogeneity in co-morbidity risk; dark blue squares = high co-morbidity risk; light blue diamonds = low co-morbidity risk.

There was no apparent overall effect of heterogeneity in co-morbidity risk on the age-curves. However, taken separately, low and high co-morbidity levels did show large effects on some outcomes suggesting that the combined effects cancel out in the scenario with heterogeneity (Figure 3). The outcomes affected were those directly affected by the action of co-morbidity risk: severe episodes (and hence direct mortality), hospital admissions, and indirect mortality. Co-morbidity does not influence acquired immunity in the model and so there were no feedback effects.

#### Probability of seeking treatment

**Figure 4:**
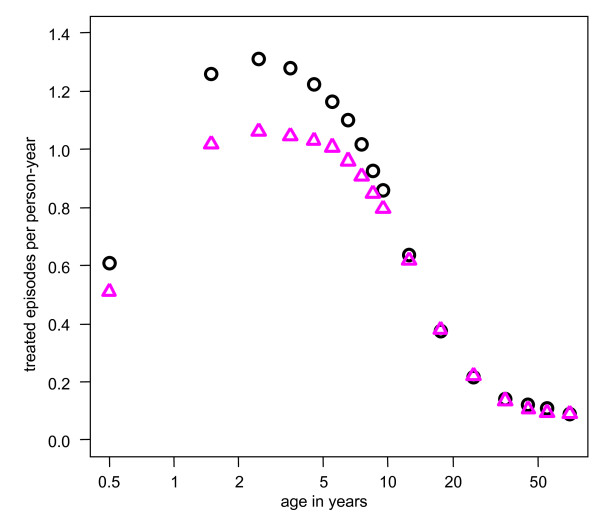
**Predicted effect of heterogeneity in the probability of seeking treatment on the incidence of first-line treatments**. Black circles = reference scenario as defined in Table 1; pink triangles = with heterogeneity in the probability of seeking treatment.

The strongest effect of heterogeneity in treatment-seeking behaviour was a reduction in the incidence of both first-line treatments and hospital admissions (Figure 4). Taken separately, low and high levels of treatment-seeking affected all outcomes: low levels lead to increased prevalence and increased incidence of episodes and deaths. When combined in the scenario with heterogeneity in treatment-seeking levels however, the overall effect was limited, with slight increases in the peak for parasite prevalence and uncomplicated episodes (Additional File 1: Table S1).

In general, the strongest effects of the single heterogeneities were reserved for outcomes directly affected by the action of the heterogeneity. Feedback via acquired immunity produced more modest effects. For some outcomes, the effects of high and low values of the heterogeneity appeared to cancel out at the community level.

### Independent and co-varying pairs of heterogeneities

The results suggest that the two independent types of heterogeneities together produce additive effects on the age-curves (Additional File 2: Table S2).

**Figure 5:**
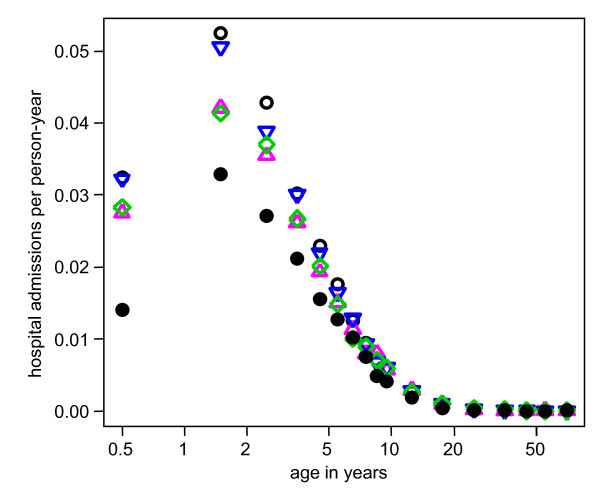
**Predicted incidence of hospital admissions with heterogeneity in risk of co-morbidity and probability of seeking treatment**. Hollow black circles = reference (Table 1); pink triangles = heterogeneity in probability of seeking treatment; blue inverted triangles = heterogeneity in risk of co-morbidity; green diamonds = heterogeneity in both treatment-seeking and co-morbidity (independent); black solid circles = co-varying heterogeneity in both co-morbidity and treatment-seeking.

**Figure 6:**
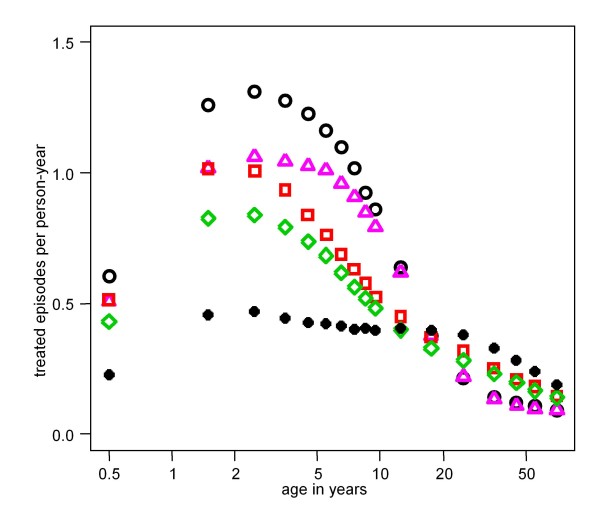
**Predicted effect of heterogeneity in transmission and treatment-seeking on the incidence of first-line treatments**. Hollow black circles = reference (Table 1); pink triangles = heterogeneity in probability of seeking treatment; red squares = heterogeneity in transmission; green diamonds = heterogeneity in both treatment-seeking and transmission (independent); black solid circles = co-varying heterogeneity in both transmission and treatment-seeking.

The results for sources of heterogeneity which co-vary were more variable. Changes in comparison to the independently varying case occurred only if the outcome measure was affected by both kinds of heterogeneity individually, either apparent or masked by high and low values cancelling out. Changes were observed in outcomes which were directly affected by the action of the heterogeneity. Striking differences compared to independent heterogeneities were produced for hospital admissions and direct mortality (co-morbidity and treatment-seeking: Figure 5) and first-line and hospital admissions (transmission and treatment-seeking: Figure 6). More modest changes were produced for other outcomes (Additional File 2: Table S2).

There were no changes for outcomes which were not affected by both heterogeneities individually. Prevalence, uncomplicated episodes and first-line treatments were unaffected by heterogeneity in co-morbidity and no changes were observed for the pairs co-morbidity and treatment-seeking, and co-morbidity and transmission. There were also no apparent changes to the age-curves for prevalence, uncomplicated and severe episodes, and direct and indirect mortality (transmission and treatment-seeking). Although both types of heterogeneity either affected these outcomes or masked effects at high and low levels, the effect of treatment is via dynamic feedback on acquired immunity. Outcomes which are not directly influenced by the action of the heterogeneities are less affected in our simulations.

The effect of heterogeneity in all three variables was simulated for completeness (Additional File 1: Table S1), however interpreting the results is difficult. In general, age-curves for outcomes affected by only two of the heterogeneities were similar to those produced by the pair of heterogeneities. Where all three had individual effects, the independent and co-varying triple heterogeneities could have effects which were very different to each other.

### The effect of heterogeneity for different transmission intensities

These simulations were repeated for different transmission intensities (EIR 1, 6 and 200) keeping the same seasonal pattern. The age-curves shift due to the effects of transmission intensity, but the direction and strength of the impact of the different types and combinations of heterogeneity were similar to those of the reference transmission of 21 infectious bites per person per year.

## Discussion

This study describes the simulated effects of heterogeneity in transmission intensity, co-morbidity risk and treatment-seeking behaviour on the age-curves of a range of measures of the burden of malaria. Although this study is specific to malaria, our findings add more generally to studies of infectious diseases indicating that it is not only the absolute level of a variable, but also the presence of heterogeneity [15,22,25,40] and co-variation [23,28], which can produce important patterns at the community level.

The results show that the different types of heterogeneity have effects on different outcomes with large effects reserved for outcomes directly affected by the action of the heterogeneity, rather than dynamic feedback via acquired immunity. Transmission heterogeneity affected the age-curves for all outcomes. The peak parasite prevalence was reduced and all age-incidence curves crossed those of the reference scenario with a lower incidence in younger children and higher in older age-groups. Heterogeneity in the probability of seeking treatment reduced the peak incidence of first-line treatment and hospital admissions. Heterogeneity in co-morbidity risk showed little overall effect, but high and low values cancelled out for outcomes influenced by its action. Independently varying pairs of heterogeneities produced additive effects. More variable results were produced for co-varying pairs, with striking differences compared to independent pairs for some outcomes which were affected by both heterogeneities individually.

These findings have implications for both the interpretation of age-curves and their use in analysis and modelling. In pointing to where the different types of heterogeneity change the shape of the age-curves, this study also indicates where there is no need for concern. Since the greatest effects of single heterogeneities were reserved for outcomes directly affected or subsequent to their action, only modest effects would be expected for outcomes less directly linked unless transmission intensity is affected. Outcomes which were unaffected by single heterogeneities do not change substantially when there are concurrent heterogenous variables, either independent or co-varying.

The results illustrate the effect of heterogeneity on the ability to infer from one outcome to another. The utility of passive case detection to estimate the burden of clinical disease depends on assumptions about the underlying distribution of treatment-seeking behaviour. Similarly, the use of clinical episodes to infer effects on mortality depends on heterogeneity in the risk of co-morbidity and treatment-seeking. Patterns of heterogeneity are also important when estimating transmission from prevalence or clinical data.

A logical consequence of the findings is that estimating parameter values by fitting a model to data from a single field site falsely assuming either homogeneity or independently varying variables is liable to produce incorrect values, as found elsewhere [15,23,25].

This study has several limitations. The adopted representation of the patterns of heterogeneity was very crude. Whilst the strength of the impact will differ, the conclusions are not dependent on the values chosen for the high and low risk groups, on the distribution of individuals between groups or on whether there is a gradual increase in risk across individuals as long as the overall values are equal in the heterogeneity and comparison scenarios. Thus, although the scenarios do not exhibit realistic patterns, the findings nevertheless provide insights on the substantive effects of different types of heterogeneity. Likewise, there was no intention to realistically portray SES. There are complex pathways between poverty, economic activity, health-care seeking and malaria [7,13,41]. The scope of this study is limited to the incorporation of simple heterogeneity in three variables which are plausibly related to SES into a model of malaria epidemiology. It was also assumed that the individual's relative levels for transmission, co-morbidity risk and treatment-seeking are constant throughout their life. This simplification ignores the impact of malaria on poverty [13,42] and mobility [43]. More sophisticated simulations may address the need to disaggregate SES [44] and identify interventions of particular benefit to the poor (such as [45]).

Assumptions were made about the likely direction of co-variation, matching high transmission to a high risk of co-morbidity and low probability of seeking treatment. In different settings and ecotypes, these directions may be reversed. Individuals are assumed to be distributed spatially at random. Spatial patterns could be better incorporated by grouping individuals into households.

Objective criteria are lacking for determining whether two age-curves differ. However, the patterns observed were clear-cut. In this study, heterogeneity in transmission had the strongest effects on the age-curves. It is plausible that this would also be the case in many real settings. However, this study does not use use a realistic patterns and degrees of heterogeneity, and so conclusions cannot be drawn about the relative importance of the three heterogeneities on the shape of the age-curves.

The average annual level of transmission was fixed to remain constant so that the effects observed were solely due to heterogeneity when compared to the reference scenario. However, heterogeneity may also alter the absolute level of transmission intensity via dynamic feedback to the infectious reservoir.

The base model used is comprehensive and individual-based providing a flexible framework for unravelling the effects of different heterogeneities. Limitations of the model components are discussed elsewhere [26,31-33,46-49]. Some assumptions are especially relevant to this study. Non-malaria co-morbidity is assumed to prompt an acute episode to lead to either a severe episode or an indirect death, and the risk of co-morbidity is assumed to be age-dependent. This is reflected in the age-curves for both the reference scenario and the inclusion of heterogeneity in co-morbidity risk. The base model assumes constant probabilities for seeking treatment within a five-day period for all individuals with either acute or severe episodes. However, treatment-seeking may be age-dependent, due to differences in the recognition of fevers or perceived need for treatment in adults, children and infants and also to the tendency for different symptoms to manifest at different ages such as severe malarial anaemia in young children [50]. The five-day time step constrains the model components for both treatment-seeking and case-management to be very simple. The time steps are currently being shortened to one day and a more sophisticated case-management model is in development.

Effects of heterogeneity on outcomes other than age-curves were beyond the scope of this paper. Heterogeneity is likely to have important effects on receptivity and elimination [15,19-21], on individual differences and equality, and on the impact of different interventions [22-24]. Some interventions may lessen the differences between individuals in a population, but the effects of reducing heterogeneity are not yet known. In addition to further simulation studies, future work should also extend the analysis of available field data to describe the pattern of heterogeneity in different settings. Innovative methods are needed to estimate sources of heterogeneity that are difficult to measure, such as for micro-transmission [25,51,52], and multivariate analysis estimating the degree and patterns of co-variation would enable more realistic scenarios to be considered.

## Conclusions

Heterogeneity is not a single entity: different types and combinations of heterogeneity lead to different effects. Interpretation and use of age-prevalence or age-incidence curves should involve consideration of the nature and extent of different heterogeneities in the populations being analysed.

## Competing interests

The authors declare that they have no competing interests.

## Authors' contributions

AR conceived the study, designed the simulations and drafted the manuscript. TS edited the manuscript. Both authors have read and approved the final manuscript.

## Supplementary Material

Additional file 1 Table S1: Summary of predicted age-prevalence and incidence curves using age and height at peak. Click here for file

Additional file 2 Table S2: Effects of pairs of heterogeneities on predicted age-prevalence and incidence curves. Click here for file
