# Supplementary material for: Interpreting malaria age-prevalence and incidence curves: a simulation study of the effects of different types of heterogeneity
Source: Malar J. 2010 May 17;9:132. doi: 10.1186/1475-2875-9-132 (PMC2888834; doi:10.1186/1475-2875-9-132)
Supplement: Additional file 1 — Table S1: Summary of predicted age-prevalence and incidence curves using age and height at peak. [file 1475-2875-9-132-S1.DOC]

**Table S1. Summary of predicted age-prevalence and incidence curves using age and height at peak**

|  | prevalence | | |  | uncomplicated  episodes | | |  | first line treatments | |  | severe  episodes | |  | hospital admissions | |  | direct  mortality | |  | indirect  mortality | |
| --- | --- | --- | --- | --- | --- | --- | --- | --- | --- | --- | --- | --- | --- | --- | --- | --- | --- | --- | --- | --- | --- | --- |
|  | age | height | |  | age | | height |  | age | height |  | age | height |  | age | height |  | age | height |  | age | height |
|  |  |  | |  |  | |  |  |  |  |  |  |  |  |  |  |  |  |  |  |  |  |
| Reference | 17.5 | 100% | |  | 4.5 | | 100% |  | 2.5 | 100% |  | 1.5 | 100% |  | 1.5 | 100% |  | 1.5 | 100% |  | 0.5 | 100% |
|  |  |  | |  |  | |  |  |  |  |  |  |  |  |  |  |  |  |  |  |  |  |
| Type of heterogeneity | | | |  |  | |  |  |  |  |  |  |  |  |  |  |  |  |  |  |  |  |
| Transmission | 17.5 | 69% | |  | 2.5 | | 78% |  | 1.5 | 78% |  | 1.5 | 73% |  | 1.5 | 73% |  | 1.5 | 64% |  | 0.5 | 79% |
| Co-morbidity | 17.5 | 100% | |  | 4.5 | | 100% |  | 2.5 | 99% |  | 1.5 | 97% |  | 1.5 | 96% |  | 1.5 | 90% |  | 0.5 | 98% |
| Treatment-seeking | 17.5 | 100% | |  | 3.5 | | 103% |  | 2.5 | 81% |  | 1.5 | 102% |  | 1.5 | 80% |  | 1.5 | 92% |  | 0.5 | 97% |
|  |  |  | |  |  | |  |  |  |  |  |  |  |  |  |  |  |  |  |  |  |  |
| Transmission – co-morbidity | | | |  |  | |  |  |  |  |  |  |  |  |  |  |  |  |  |  |  |  |
| Independent | 17.5 | 70% | |  | 2.5 | | 78% |  | 1.5 | 77% |  | 1.5 | 73% |  | 1.5 | 72% |  | 1.5 | 62% |  | 0.5 | 93% |
| Co-varying | 17.5 | 68% | |  | 2.5 | | 76% |  | 1.5 | 77% |  | 1.5 | 93% |  | 1.5 | 94% |  | 1.5 | 82% |  | 0.5 | 114% |
|  |  |  | |  |  | |  |  |  |  |  |  |  |  |  |  |  |  |  |  |  |  |
| Transmission – treatment-seeking probability | | | | | | | |  |  |  |  |  |  |  |  |  |  |  |  |  |  |  |
| Independent | 17.5 | 72% |  | | | 2.5 | 81% |  | 2.5 | 64% |  | 1.5 | 79% |  | 1.5 | 63% |  | 1.5 | 73% |  | 0.5 | 93% |
| Co-varying | 17.5 | 66% |  | | | 1.5 | 82% |  | 2.5 | 36% |  | 1.5 | 82% |  | 1.5 | 37% |  | 1.5 | 91% |  | 0.5 | 93% |
|  |  |  |  | | |  |  |  |  |  |  |  |  |  |  |  |  |  |  |  |  |  |
| Co-morbidity - treatment-seeking probability | | | | | | | |  |  |  |  |  |  |  |  |  |  |  |  |  |  |  |
| Independent | 17.5 | 101% |  | | | 3.5 | 103% |  | 2.5 | 82% |  | 1.5 | 103% |  | 1.5 | 79% |  | 1.5 | 101% |  | 0.5 | 101% |
| Co-varying | 17.5 | 100% |  | | | 3.5 | 103% |  | 2.5 | 84% |  | 1.5 | 113% |  | 1.5 | 63% |  | 1.5 | 125% |  | 0.5 | 114% |
|  |  |  |  | | |  |  |  |  |  |  |  |  |  |  |  |  |  |  |  |  |  |
| Triple |  |  |  | | |  |  |  |  |  |  |  |  |  |  |  |  |  |  |  |  |  |
| Independent | 17.5 | 71% |  | | | 2.5 | 81% |  | 2.5 | 64% |  | 1.5 | 79% |  | 1.5 | 65% |  | 1.5 | 74% |  | 0.5 | 77% |
| Co-varying | 12.5 | 64% |  | | | 1.5 | 80% |  | 1.5 | 37% |  | 1.5 | 105% |  | 1.5 | 37% |  | 1.5 | 109% |  | 0.5 | 123% |

Age in years (mid-point of age group)

Height of peak as a percentage of the reference peak height
