# Supplementary material for: Interpreting malaria age-prevalence and incidence curves: a simulation study of the effects of different types of heterogeneity
Source: Malar J. 2010 May 17;9:132. doi: 10.1186/1475-2875-9-132 (PMC2888834; doi:10.1186/1475-2875-9-132)
Supplement: Additional file 2 — Table S2: Effects of pairs of heterogeneities on predicted age-prevalence and incidence curves. [file 1475-2875-9-132-S2.DOC]

**Table S2. Effects of pairs of heterogeneities on predicted age-prevalence and** incidence curves

| Pair | Outcomes | Effect of individual heterogeneities | Effects of pair varying independently | Effects of pair when co-varying |
| --- | --- | --- | --- | --- |
| Transmission – treatment-seeking behaviour | Prevalence  Uncomplicated  Severe  Direct mortality  Indirect mortality | Transmission heterogeneity reduced peak prevalence and lead to a cross-over^‡^ for uncomplicated and severe episodes. Treatment-seeking heterogeneity had little effect overall, results for high and low values appear to cancel out. | Similar to effect for heterogeneity in transmission alone† | Similar to independently varying sources of heterogeneity |
|  | First-line treatments  Hospital admissions | Transmission heterogeneity leads to a cross-over^‡^ Treatment-seeking leads to a reduction in peak incidence. | Peak incidence was reduced, the amount suggesting an additive effect of the two single heterogeneities (Figure 6). | Large decreases in the peak incidence of first-line treatments and hospital admissions (Figure 6). |
|  |  |  |  |  |
| Co-morbidity – treatment-seeking behaviour | Prevalence  Uncomplicated  First-line treatments | Heterogeneity in co-morbidity showed little effect. Heterogeneity in treatment-seeking had little overall effect, low and high values cancelled out. | Similar to heterogeneity in treatment-seeking alone† | Similar to independently-varying heterogeneities. |
|  | Severe  Hospital admissions  Direct mortality  Indirect mortality | Heterogeneity in co-morbidity and treatment-seeking showed little overall effect since results from high and low values cancelled each other out. | Similar to heterogeneity in treatment-seeking alone† | Large decrease for hospital admissions. Small increases in peak heights for severe episodes, and direct and indirect mortality (Figure 5). |
|  |  |  |  |  |
| Transmission – co-morbidity risk | Prevalence  Uncomplicated  First-line treatments | Heterogeneity in transmission reduced peak prevalence and lead to a cross-over^‡^ for uncomplicated episodes and first-line treatments. Heterogeneity in co-morbidity risk had little effect. | Similar to heterogeneity in transmission alone† | Effect remains similar to independently-varying heterogeneities. |
|  | Severe  Hospital admissions  Direct mortality  Indirect mortality | Transmission heterogeneity lead to a cross-over^‡^ Heterogeneity in co-morbidity showed little overall effect but high and low values cancelled each other out. | Similar to heterogeneity in transmission alone† | Modest increase in peak compared to independently-varying heterogeneities. |

^†^ Second heterogeneity had no overall effect as an individual heterogeneity

^‡^Incidence was reduced for younger ages compared to the reference scenario and increased for older age groups
